# The Protective Role of Maternal Immunization in Early Life

**DOI:** 10.3389/fped.2021.638871

**Published:** 2021-04-28

**Authors:** Bianca Cinicola, Maria Giulia Conti, Gianluca Terrin, Mayla Sgrulletti, Reem Elfeky, Rita Carsetti, Ane Fernandez Salinas, Eva Piano Mortari, Giulia Brindisi, Mario De Curtis, Anna Maria Zicari, Viviana Moschese, Marzia Duse

**Affiliations:** ^1^Department of Maternal and Child Health and Urological Sciences, Policlinico Umberto I, Sapienza University of Rome, Rome, Italy; ^2^Department of Experimental Medicine, Sapienza University of Rome, Rome, Italy; ^3^Department of Molecular Medicine, Sapienza University of Rome, Rome, Italy; ^4^Pediatric Immunopathology and Allergology Unit, University of Rome Tor Vergata, Policlinico Tor Vergata, Rome, Italy; ^5^Ph.D. Program in Immunology, Molecular Medicine and Applied Biotechnology, University of Rome Tor Vergata, Rome, Italy; ^6^Department of Clinical Immunology, Royal Free Hospital, London, United Kingdom; ^7^Infection, Immunity & Inflammation Department, Institute of Child Health, University College London (UCL), London, United Kingdom; ^8^Diagnostic Immunology Research Unit, Multimodal Medicine Research Area, Bambino Gesù Children's Hospital, IRCCS, Rome, Italy; ^9^Microbiology and Diagnostic Immunology Unit, Bambino Gesù Children's Hospital, IRCCS, Rome, Italy; ^10^Department Saint Camillus International University of Health and Medical Sciences, Rome, Italy

**Keywords:** maternal immunization, vaccination, pregnancy, immune system, neonate

## Abstract

With birth, the newborn is transferred from a quasi-sterile environment to the outside world. At this time, the neonatal immune system is inexperienced and continuously subject to a process of development as it encounters different antigenic stimuli after birth. It is initially characterized by a bias toward T helper 2 phenotype, reduced T helper 1, and cytotoxic responses to microbial stimuli, low levels of memory, and effector T and B cells and a high production of suppressive T regulatory cells. The aim of this setting, during fetal life, is to maintain an anti-inflammatory state and immune-tolerance. Maternal antibodies are transferred during pregnancy through the placenta and, in the first weeks of life of the newborn, they represent a powerful tool for protection. Thus, optimization of vaccination in pregnancy represents an important strategy to reduce the burden of neonatal infections and sepsis. Beneficial effects of maternal immunization are universally recognized, although the optimal timing of vaccination in pregnancy remains to be defined. Interestingly, the dynamic exchange that takes place at the fetal-maternal interface allows the transfer not only of antibodies, but also of maternal antigen presenting cells, probably in order to stimulate the developing fetal immune system in a harmless way. There are still controversial effects related to maternal immunization including the so called “immunology blunting,” i.e., a dampened antibody production following infant's vaccination in those infants who received placentally transferred maternal immunity. However, clinical relevance of this phenomenon is still not clear. This review will provide an overview of the evolution of the immune system in early life and discuss the benefits of maternal vaccination. Current maternal vaccination policies and their rationale will be summarized on the road to promising approaches to enhance immunity in the neonate.

## Introduction

Despite significant advances in child survival in the last few decades, infectious diseases continue to be among the main causes of morbidity and mortality, especially in the neonatal period (1). Newborns are at increased risk of infections because their distinct immune system is not always able to mount an efficient protective immune response against pathogens (2).

Extended vaccination programs worldwide have significantly improved child survival by preventing infections such as polio, pertussis, smallpox, and measles (3, 4). However, when it comes to neonatal immunization, there are just a few vaccines licensed for administration in the first days of life (5). Several factors might affect programming of the immune system in early life and immune response might differ at neonatal and later ages. Thus, scarce knowledge and awareness of risks and benefits contribute to low neonatal immunization (6).

Maternal immunization has been recognized and recommended as a public health strategy to protect the mother, fetus, and infant from infections. Maternally derived pathogen specific antibodies represent a tool to protect the vulnerable infants until their immune system can adequately respond to vaccinations or infections (7). In fact, maternal antibodies are passively transferred throughout the placenta and later in colostrum and breast milk, ready to combat infections in early life (8). Optimal concentration of transplacentally transferred maternal antibodies, and the exact timing of maternal immunization, are still a matter of debate (9, 10). An open issue remains the so-called “immunology blunting,” i.e., the phenomenon by which maternal Immunoglobulin G (IgG) antibodies may dampen the response of the child to vaccination (11). On the other hand, it has been suggested that the mother may also pass immune cells to the child by placental transfer. Maternal cells may help the development of the fetal and neonatal immune system (12).

Herein, we will review some aspects of maternal, fetal and neonatal immune systems in the context of maternal immunization and highlight the current status of vaccination in pregnancy. Taken together, these data provide a framework to update our current understanding and to open new vaccine avenues in the field of immunization in pregnancy and the young infant.

### Search Strategy

To retrieve information for this review, a PubMed based research was conducted using the medical subject heading database terms “vaccination” OR “immunization” AND “pregnancy.” In addition, maternal, fetal, immune system, placenta, neonate were used as search terms. We also included the currently allowed, contraindicated and in development vaccines for pregnant women, as well as the recent evidences on COVID-19. Filters for humans, any publication date and articles in English language were applied. For this narrative review, evidence was included from randomized clinical trials, original research and observational studies, case series, position statements, systematic reviews, meta-analysis studies, and selected reviews addressing the covered questions or cross-references from these publications.

## Maternal and Fetal Immune System

### Maternal Immune System and the Maternal-Fetal Interface

Early in pregnancy, the maternal immune system undergoes a timely regulated remodeling to allow the implantation, preservation, and growth of the semi-allogenic fetus while protecting against pathogens (13–15). Maternal and fetal immune systems establish a cooperative status (16) depending on a delicate balance between anatomic, endocrine, metabolic, and microbiome factors (17). Of note, in pregnant women, the ability to mount an adequate antibody response and immunologic memory following an infection or vaccination is not affected (18–20).

Main changes in the maternal immune system can be summarized into three phases: the 1st trimester requires a strong proinflammatory state to guarantee the implant of the blastocyst in the uterus. This process implies the break of the epithelial lining of the uterus, damage of the endometrial tissue and a rearrangement of the endothelium and vascular smooth muscle of the maternal blood vessels to establish an adequate placental–fetal blood supply (21). All these activities require an inflammatory environment to secure the adequate repair of the uterine epithelium and the removal of cellular debris; in the 2nd and 3rd trimester, the anti-inflammatory response prevails, allowing tolerance of the semi-allogenic fetus, its rapid growth and development. Finally, the switch to a new proinflammatory state favors the cascade of events that synergistically lead to parturition (22, 23). Specific cell populations mediate the transition from the pro- to the anti-inflammatory state and viceversa. First, T lymphocytes take part through a shift from a type 1 T helper lymphocytes (Th1) response, oriented toward cell-mediated immunity, toward a type 2 T helper lymphocytes (Th2) response, which favors humoral immunity by stimulating B cells to increase the production of maternal antibodies (24). Th2 lymphocyes are fundamental for the mother-child immunological tolerance up to the time of delivery, suppressing cytotoxic T cells activity (25). Moreover, T regulatory cells (Tregs), a sub-type of CD4+ T cells that express CD25, increase in pregnancy and contribute to the maintenance of immunological tolerance to the fetus by actively suppressing self-reactive lymphocytes (26, 27).

In pregnancy, the maternal immune barrier to infections enlists a combination of signals and immune modulators originating from the fetoplacental dyad (28), including the placenta and decidua. In fact, the placenta is an active site of innate immune response capable of reacting to pathogens by the release of antimicrobial peptides and cytokines (13, 16). Nevertheless, a second important site of innate immune responses is localized at the decidual layer. The maternal-fetal interface represents the direct contact between the embryo and the mother and it is populated by fetal trophoblast cells, maternal decidual stromal cells (DSCs), and decidual immune cells (DICs) (29). The decidua is an active site of chemokine synthesis throughout gestation, attracting neutrophils, natural killer cells, dendritic cells, and macrophages (17). However, the transfer of innate inflammatory mediators from the mother to the fetus is less well-understood (16). Finally, early in the second trimester, the maternal immune system protects the fetus by passive transfer of maternal antibodies (IgG) and maternal immune cells across the placenta (30).

### Fetal and Neonatal Immune System

Recent technological advances have provided critical information in the field of early life human immunology with its timely and spacely development (31). The human immune system starts developing after the first 2–3 weeks of fetal life, when hematopoiesis and generation of pluripotent and self-renewing hematopoietic stem cells (HSC) begin in the yolk sac and the aorta-gonad- mesonephros region of the embryo (32). Subsequently, HSC migrate to the fetal liver, representing the major hematopoietic site during fetal life, as localization into the bone marrow occurs by week 20 (33). T cell differentiation and maturation occur in the thymus. During embryogenesis, immune cells also inhabit peripheral organs, including skin, intestine, kidney, and lung, and participate in the respective organ environment (34).

Innate immune cells are the first to emerge: granulocytes, NK cells and lymphocyte precursors are detected in the fetal circulation between gestational week (GW) 8–10 (35). From GW 8, HSCs migrate to the thymic rudiment and, by GW 16–20, mature T cells are released into the peripheral blood (36). Lymphoid precursor cells develop into B-lymphocytes with subsequent functional maturation in secondary lymphoid tissue (e.g., lymph nodes and spleen) toward the end of the first trimester (37). Thus, during the second trimester, immune cells, predominantly lymphocytes, grow in number, mature, and differentiate. Toward the end of the third trimester, the fetal immune system is functional and capable of producing a response (38).

Diverse immune cell types develop and mature at various gestational stages, which is necessary to establish feto-maternal tolerance and functional responses according to specific demands. The fetal and neonatal T-cell response is shifted toward the suppression of cytotoxicity: with a strong Th2 polarization, a dominant anti-inflammatory cytokine profile (39) and Tregs dominating the fetal circulation, suppressing reactivity to non-inherited maternal antigens (40).

At birth, the infant's response to Toll-like receptor (TLR) (i.e., innate immune response) is characterized by high production of IL-10, IL-6 and IL-23, which induce IL-17-producing helper T cells (Th17 cells) (41). The predominance of a Th17-like pattern, combined with considerable IL-10 production, may contribute to diminished T helper type 1 (Th1) responses, resulting in greater susceptibility to intracellular infections and diminished vaccine responses during infancy (42).

Since delivery, exposure to antigens and environmental challenges during infancy and childhood will promote further adaptation and expansion of the immune system (43). The exposure to the antigen rich world during the first weeks after birth leads to a massive increase in lymphocytes in a way that is largely independent of gestational age (GA) at birth. B and T cells are abundant at birth, followed by a gradual decrease over the first years of life until adulthood. As expected, the newborn presents with a lower number of memory B cells compared to older ones, reflecting the lack of exposure to foreign antigens during fetal life (33). The frequency of switched and non-switched memory B cells increases slowly with age and reaches adult levels in children 10–15 years of age (44).

Briefly, the human immune system follows a precise developmental trajectory with extraordinary plasticity in response to the different demands. However, after antigen exposure, specific protective immunity takes time; thus maternal immunization represents a great opportunity to reduce the risk of disease in the mother, fetus, and infant (30).

## How Maternal Vaccination Impacts the Immune System in Early Life

### Transfer of Maternal Antibodies Through the Placenta and Breast Milk

**Figure 1:**
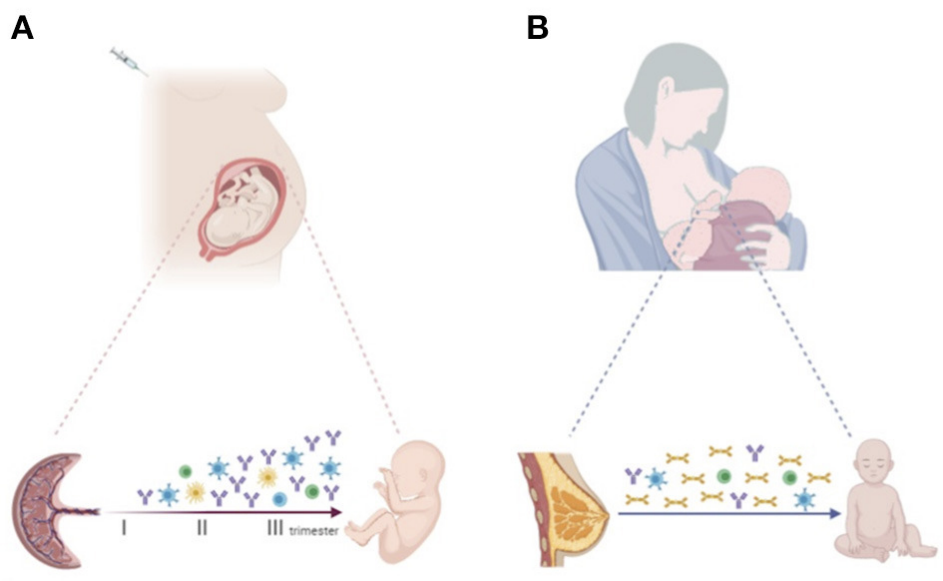
**(A)** Progressive increase of vaccine-specific antibody production and transplacental transfer after maternal immunization during pregnancy. Maternal vaccination may also impact the transfer of maternal immune cells to the offspring, priming the developing fetal immune system. **(B)** Breastfeeding enables the transfer of vaccine-specific maternal antibodies, mainly sIgA, and immune cells secreted through colostrum and breast milk to the infant. Created with BioRender.com.

Immunization of the mother during pregnancy increases vaccine-specific antibodies to ensure effective protection not only of the mother, but also the offspring in first months of life (19) (Figure 1A). The concentration of maternal antibodies in the serum of the neonate determines the effectiveness of protection. Understanding the mechanisms by which IgGs are transferred across the placenta is important to develop optimal maternal and infant immunization strategies and make decisions about the timing of vaccination in pregnancy (45, 46).

The transfer mechanisms depend on various factors, including the syncytiotrophoblast and the capillary barrier. IgGs are selectively transported from the maternal blood to the fetus via neonatal Fc receptor (FcRn) expressed in syncytiotrophoblast of the placenta (47). FcRn is a major histocompatibility complex (MHC) class I-related Fc receptor involved in the bidirectional transcytosis of IgG and IgG immune complexes across various human epithelia. It binds the Fc fragment of Ig antibodies at acidic pH (48). In maternal blood, with a physiological pH of 7.4, IgG cannot bind the FcRn at the apical side of the syncytiotrophoblasts and need to be transported by endocytosis. In the acid environment of endosomes, FcRn-IgG complexes are then carried to the fetal side of the syncytiotrophoblast, where IgG is released upon exposure to normal pH (30). IgG subsequently crosses the villous stroma and fetal capillary endothelium and enters fetal circulation, although this mechanism is not fully understood (49).

Several factors affect the IgG transfer from the mother to the fetal circulation, i.e., the amount of FcRn expressed by syncytiotrophoblasts and total maternal IgG levels. Indeed, it was observed that when the amount of maternal IgG reaches a level of 15 g/L, FcRn is saturated and maternal antibodies stop to be transferred (50).

Placental transfer of IgG also depends on gestational age. The process starts during the first trimester of pregnancy and increases exponentially over time (51). Most of the IgG transfer occurs after 28 weeks of gestation (52). As pregnancy progresses and the placenta grows in cell mass, the expression of FcRn might increase with relatively higher antibody transport rate (53). Although in preterm infants, the reduced period of gestation leads to a lower and less efficient IgG placental transfer, several studies support the efficacy of early maternal vaccination in reducing the risk of infections at birth (54, 55). Recent UK guidance recommended pertussis immunization from 20 GW to optimize protection of preterm infants (56).

Different specificities of IgG antibodies are passively transferred to the newborn, depending on maternal immunological experience, and on the type of vaccine administered in pregnancy (57). The transfer efficacy through the placenta depends on the IgG subtype affinity for the FcRn receptor (58). IgG1 is the subtype of antibodies transferred most efficiently to the fetus, followed by IgG4, IgG3, and IgG2 (52). This differential ability of immunoglobulin to be transferred is important in the context of vaccination in pregnancy. While IgG1 and smaller amounts of IgG3 and IgG4 are induced predominantly by vaccines containing protein antigens, such as tetanus, dipheteria, pertussis (Tdap), IgG2 with some IgG1, are induced predominantly by vaccines containing polysaccharide antigens, such as those against Haemophilus influenzae type b or Neisseria meningitidis (59, 60).

Previous maternal infections are another factor that can impact the specific and amount of maternal IgG antibodies to be transported across the placenta (45), as reported in mothers infected by human immunodeficiency virus-1 (HIV-1) or malaria (61–63). To provide greater protection to the infant, specific immunization programs should be developed in childbearing age or in pregnant women with chronic infections (60).

Breastfeeding represents another mechanism by which the mother protects the neonate against pathogens in early life. Indeed, maternal secretory IgA (sIgA) and, to a lesser extent IgG and IgM, are secreted in the colostrum and breast milk and seed the luminal tract of the newborn, conferring an immunological benefit (64). In particular, sIgA provides defense at the mucosal level against pathogens of the gastrointestinal and respiratory tract through direct neutralization, inactivation, and prevention of adherence to epithelial cells of toxins and other virulence factors (65).

Several studies on the protective role of breast milk in women vaccinated during pregnancy identified a high amount of vaccine-specific sIgA in the breast milk samples up to several weeks postpartum (66). Not surprisingly, a lower incidence of respiratory illness with fever episodes in young infants of influenza-vaccinated mothers was also reported (67).

Of note, different maternal immune cell populations, including dendritic cells, macrophages, natural killers (NK), memory B and T cells are also detected in breast milk. It has been suggested that these cells may modulate the developing neonatal and infant immune system (68) (Figure 1B).

### Blunting Effect of the Maternal Immune System

Despite evidences of the protective role of maternal IgG antibodies, there are concerns about their possible interference with the infant antibody response to the vaccines administered in the first months of life. It has been suggested that the presence of maternal antibodies might result in a decreased antibody production by the infant after vaccination and, consequently, in decreased protection for the child (11). This phenomenon, called “blunting,” has been associated not only to the antibodies transferred by the mother to the fetus after vaccination, but also to those generated in response to natural infection before or during pregnancy. Immune blunting has been observed in several studies regarding tetanus, diphtheria, pertussis, influenza, measles, and mumps maternal vaccination (69–71), but results are still controversial (72–74), etiopathogenesis and clinical relevance are still to be clarified (50, 75). Indeed, low antibody levels post-vaccination do not necessarily imply a low degree of protection (76). Children born to immunized mothers and vaccinated against diphtheria, tetanus, Polio, Hep B, and Hib had lower antibodies levels than children of non-vaccinated mothers. However, specific antibodies reached the seroprotective levels for all antigens (73, 77). In addition, no evidence of a clinically significant effect of blunting has been found in the United States (US) and the United Kingdom (UK), where maternal Tdap vaccination has been implemented since 2011 (US) and 2012 (UK) (78, 79).

Protection induced by vaccines may be related not only to antibody concentration, but also to their functional characteristics, such as avidity and neutralization potential, and to the generation of memory B cells, all aspects not extensively assessed yet (77). Finally, the effect of maternal antibodies on cellular immune responses after infant vaccination is another interesting aspect to consider. A recent review reporting five studies on humans concluded that pre-existing maternal antibodies do not affect infants' cellular immune response after primary vaccination (70). Specific T cell responses have been observed after immunization in the presence of maternal antibodies (71, 80). Also, maternal antibodies could prime the infant immune system to develop a stronger and long life cellular response, the so-called “prime-boost” effect (81, 82).

### Transfer of Maternal Cells Through the Placenta

The fetal immune system can be primed and responds to foreign antigens during pregnancy. Increasing evidence suggests that maternal infection or vaccination during gestation may shape and train the fetal immune system even in the absence of fetal infection, affecting infants' immune responses to pathogens and vaccines during the extrauterine life (83) (Figure 1A).

The exact mechanisms by which the fetal immune system can be primed by antigen in the absence of fetal infection remain unclear. A possible explanation is the vertical transmission of a low level of antigens from the mother to the fetus. A second hypothesis is that maternal cells carrying the antigen or antigen-loaded microvesicles cross the placental barrier (83, 84). Actually, maternal cells are found in human fetal tissues from the second trimester of pregnancy onwards (85, 86). This natural phenomenon is referred to as microchimerism (Mc). It is defined as the presence in an organism of a small number of cells or amount of DNA deriving from a genetically different individual. The Mc origins from bi-directional cell exchange during pregnancy (87).

Among the accepted functions of Mc, that is worth mentioning is promoting feto-maternal tolerance and improving the outcome of future pregnancies. Feto-maternal tolerance is the process that allows fetal and maternal cells and tissues to avoid immune rejection and coexist. Fetal immune components, exposed to maternal tissues, tolerate foreign non-inherited maternal antigens (NIMAs). On the other hand, mothers during pregnancy, encompass genetically foreign paternal antigens expressed by the developing fetus. This coincides with a bidirectional transfer across the placenta of cells that seed both in maternal and fetal tissues. The presence of maternal microchimeric cells in the fetus favors the generation of fetal Tregs, which are generated *in utero* against NIMAs expressed by maternal cells (85) and assure fetal tolerance suppressing fetal T cells functions.

Besides promoting tolerance, Mc might be crucial to prepare the fetal immune system to face the pathogens after birth. Despite the predominant tolerogenic context, the existence of fetal memory T cells has been reported in the fetal spleen (88) and gut (89), as well as in the cord blood (90). The generation of memory T cells requires fetal exposure to foreign antigens presented by antigen-presenting cells (APC). It has been supposed that maternal APC loaded with antigen may pass the placenta and prime the fetal immune system.

Fetally memory T cells have been also found in uninfected children born to mothers infected with human immunodeficiency virus (HIV) (91), hepatitis B virus (HBV) (92), hepatitis C virus (HCV) (93), or plasmodium (94). In endemic regions, it has been observed that maternal infection by helminths, including filariasis (95), schistosomiasis (96), onchocerciasis (97), and ascariasis (98) was associated with fetal lymphocytic responses and the consequent production of specific immunoglobulins. If fetal immune cells respond to antigens of infectious agents carried by the mother, it seems reasonable that the fetal immune system may also respond to maternal antigen exposure to pathogens. Interestingly, maternal tetanus toxoid immunization led to IgM anti-tetanus antibodies in the blood of children before neonatal immunization with tetanus vaccine, showing that vaccine antigen is available and that it activates the fetal immune system (99). Of note, influenza virus-specific T cells were detected in cord blood cells of neonates whose mothers received vaccination during pregnancy (100).

The exposure to antigens mediated by maternal cells *in utero* might affect the development of fetal innate immune cells as well, such as macrophages and monocytes. Not surprisingly, fetal programming of monocytes is functionally different from the adult one. It has been demonstrated that in fetal monocytes stimulation with IFN-γ, IL-6, or IL-4 generate distinct JAK/STAT signaling responses, thus triggering innate responses and antimicrobial activities rather than promoting a strong adaptive and potentially harmful inflammatory response (101). Fetal monocytes can mount a more primitive, but potentially protective, innate antimicrobial response, which may also optimize the fetus and newborn's chance of successfully combating a microbial invader.

These observations add important information to understand the potential of vaccination in pregnancy including the ability to modulate the development of the fetal innate immune system (see paragraph “Future directions” on live-attenuated vaccines).

The timing of maternal vaccination can influence the transplacental transfer of vaccine antigens and the subsequent fetal immune response. In other words, the gestational age when the vaccine is administered may have an impact on priming. A study on allergen exposure in pregnancy suggested that T-cell priming with inhalant and food allergens may occur starting from 22 weeks' gestation (102).

Fetal exposure to pathogens-related antigens during pregnancy might lead to acquired protective immune responses in the offspring. However, there is still concern that it may also cause immune tolerance, increasing infants' susceptibility to both homologous and unrelated pathogens (103, 104). Non-replicating modified vaccinia Ankara was associated with reduced responses to unrelated pathogens, indicating some degree of induced tolerance (102); Diphteria-Tetanus-Pertussis (DTP) has also been shown to induce immune tolerance to unrelated antigens 3 months after vaccination, with immuno-tolerance partly restored by concurrent or subsequent BCG vaccination (105). T cell anergy and a rise in Tregs might be responsible for immune tolerance (106). Tregs have been shown to suppress antigen-specific immune responses to malaria in infants born to mothers with infection during pregnancy (107).

## Maternal Vaccination in Clinical Practice

### Current Recommendations

#### Influenza Vaccine During Pregnancy and Breastfeeding

Pregnant women have a higher risk than normal population to develop severe and life-threatening complications due to influenza (Flu) virus infections (25, 108), with an increased incidence of cardiorespiratory involvement. This could be partially explained by the physiological cardiopulmonary adaptation during pregnancy due to the welcoming fetus. Moreover, immunological alterations, with reduction of T-helper 1 cell-mediated cytotoxic T activity and B cell expansion, could also play a role (109).

Flu infection is also associated with increased risk of poor pregnancy outcomes with a higher incidence of stillbirth, preterm birth and low birth weight. During the 2009 H1N1 influenza pandemic, as reported by Callaghan et al. the pregnancy-related mortality ratio for confirmed or probable H1N1 pandemic was equal to 2.2 per 100.000 live births (110, 111). During the same period, an increased prevalence of intensive care unit requests, low Apgar scores, fetal demises, preterm births, neonatal deaths, and low birth weights has been associated with the H1N1 pandemic (112). Moreover, infants younger than 6 months of age are at risk of severe complications during flu infection and for them, an effective flu vaccine is not yet available. In fact, due to the low immunogenicity of available influenza vaccine preparations, none of these vaccines is licensed for them (25).

Under this scenario, it is not surprising that the interest for flu vaccine during pregnancy has always been high. The usage of inactivated influenza vaccines (IIV) has been reported in the US since the 1950s, with an optimal spectrum of safety. Firstly, licensed in 1997 for pregnant women in the second or third trimester, IIV has then been recommended in 2004 for all pregnant women during flu season (113). Following data of 2009 H1N1 influenza pandemic, pregnant women have been identified as one of the highest priority groups under the immunization program and, since 2012, the recommendation to use IIV has been extended by the World Health Organization (WHO) to all pregnant women, regardless of their time of gestation (114). Similarly, both the Advisory Committee on Immunization Practices (ACIP) and the American College of Obstetricians and Gynecologists (ACOG) recommend IIV for all pregnant women at any time of gestation, as well as for all women planning to get pregnant or in the postpartum phase during flu season (115–117).

Conversely, Live Attenuated Influenza Vaccine (LAIV) is contraindicated during pregnancy and data on the use of recombinant influenza vaccine (RIV) in pregnant women are limited.

Worldwide, the efficacy of the flu vaccine for both the mother and fetus has been demonstrated. In a large Norwegian study including more than 117.000 pregnant women, a significant decrease in flu diagnosis and a relevant—although not statistically significant—reduction of fetal death risk has been reported (118). Another study, conducted on 2.310 South African pregnant women, 194 of them affected by human immunodeficiency virus (HIV) infection, has shown a decrease of confirmed flu infections in HIV and non-HIV infected women (119).

No association between IIV and adverse pregnancy events, such as congenital malformations, spontaneous abortion or preterm birth, has been reported (120). Conversely, flu vaccine administration in pregnant women has been associated with a lower rate of pregnancy-related adverse events such as preterm birth, low birthweight or fetal death (118, 121).

Thus, flu vaccine administration in pregnancy and related, specific maternal antibodies, also protects the newborns during the first 6 months of life, with an estimated reduction of 72% of laboratory-confirmed influenza hospitalizations at that age (122, 123). Another study conducted by Benowitz et al. has shown that maternal immunization against flu reduces of 91.5% influenza-related hospitalizations in children < 6 months of age (124).

Finally, IIV could be administered at postpartum or while breastfeeding in women not previously vaccinated during pregnancy (120).

#### Tdap (Tetanus, Diphtheria, Pertussis) Vaccine During Pregnancy

Nowadays, tetanus and diphtheria are rare in industrialized countries thanks to the routine immunization programs. Due to reduced hygiene conditions, these diseases are common in low and middle-income countries, particularly in pregnant women and their offspring. The possibility to eradicate tetanus is currently unrealistic because of its intrinsic characteristics: high spread in the environment, high resistance to antimicrobial measures, and possible transmission by an open wound.

Unvaccinated pregnant women and their newborns—when unprotected by passive immunity—are at higher risk of severe complications due to tetanus disease. Also, infants born from unvaccinated mothers are unprotected against tetanus during the first months of life (25). The use of unsterile instruments and poor hygiene conditions are the main risk factors (111).

This underlines the importance of preventive measures such as vaccination programs combined with improvement of hygiene conditions and pre, peri, and postnatal care and post-exposure prophylaxis in unsafe conditions (125).

Since 1989, due to severe epidemiologic data—with 787.000 newborns death of neonatal tetanus in 1988 and an estimated annual global mortality of 6.7 per 1.000 live births—WHO promoted the Maternal and Neonatal Tetanus Elimination (MNTE) program, to reduce maternal and neonatal tetanus (MNT) so that it would no longer be a public health problem.

The MNTE initiative involves the implementation of both maternal anti-tetanus immunization and hygiene conditions during pre, peri and postnatal care practices. In 2015, thanks to MNTE efforts, a decrease of 96% in neonatal tetanus were reached, with 34.000 related deaths in newborns were reported during that year. Further progress has been made with an even more significant global reduction of deaths due to neonatal tetanus, equal to 25.000 cases in 2018. Data relative to the first seven months of 2019 have shown that neonatal tetanus has yet to be eliminated in 12 countries (126).

In order to reach a long-term immunity for tetanus, vaccine booster doses are required (125, 127). According to WHO recommendation, in previously unvaccinated pregnant women or if the immunization status is unknown, two doses of a tetanus toxoid-containing vaccine (TT-CV) are recommended, the second dose being 1 month after the first dose and at least 2 weeks before childbirth. A third dose should be administered 6 months after the second one to protect for at least 5 years. For these women who have received the first tetanus vaccine during pregnancy, two further doses should be administered after the third dose, in two subsequent years, or during two subsequent pregnancies. Conversely, in women with a previous history of 1–4 doses of tetanus vaccine administration, only one dose of TT-CV should be given (128).

While tetanus and diphtheria are uncommon in industrialized countries, pertussis is still globally endemic. Infants in the first month of life are at higher risk of severe complications, including pneumonia, apnea, encephalopathy, and multiorgan failure due to pertussis infection. Pertussis could also be fatal, mainly at this early age (109). Despite the effectiveness of the pertussis vaccine—licensed in the 1950'—in reducing morbidity and mortality, several outbreaks have occurred in different countries (129, 130).

During the 2010 pertussis outbreak in California, more than 9.000 cases have been reported, with 10 deaths, all in infants < 2 months of age, 9 of which were healthy before the infection (131). Another pertussis resurgence occurred in 2012 in the United Kingdom (UK), resulting in the death of 14 infants (25).

To reduce the burden of whooping cough in infants, both in developing and industrialized countries, WHO recommended immunization program in pregnant women. Of note, the pertussis vaccine is not administered as a mono-component but only in combination with tetanus and diphtheria.

In their statements, ACIP and WHO recommended providing one dose of Tdap to all pregnant women at each pregnancy, regardless of previous vaccine history (132). Tdap should be administered at 27–36 weeks of gestation, although it could be given at any time during pregnancy. Women who were not vaccinated during pregnancy should receive Tdap vaccine postpartum.

The choice of the last trimester as the best timing option for Tdap immunization depends on the highest concentration of maternal specific IgG and on the most efficient placental transport after 34 weeks of pregnancy as previously mentioned (109).

The safety of Tdap vaccination during pregnancy has been demonstrated across different studies. Among a cohort of 123.494 pregnant women, of whom 21% have been vaccinated with Tdap, there was no association between Tdap vaccine and adverse birth events such as preterm delivery and low birth weight (133).

### Additional Vaccines in Special Circumstances

Additional vaccines can be considered to protect against infectious diseases in the case of traveling to at-risk areas or close positive contacts. Anti-hepatitis A and B, pneumococcus and meningococcus vaccines are an example of this condition (25). In the same way, further vaccines such as those against tick-borne or Japanese encephalitis should be administered if the risk of these infections is high and/or in endemic areas during disease re-surgence (6, 25).

### Vaccine Contraindications

Live attenuated vaccines (LAV), including measles, mumps, rubella and varicella vaccine (MMR or MMRV), are generally contraindicated in pregnant women, as a precautionary measure, because of the theoretical risk of perinatal infection. LAV are burdened by the risk of transplacental vaccine-virus/bacterial infection. Furthermore, data regarding LAV safety during pregnancy are limited due to the exclusion of pregnant women from clinical trials (134).

However, data from accidental vaccination with MMRV in pregnant women have shown that the risk of adverse pregnancy-related events is rare (109). Evidence regarding other LAVs is lacking (135).

#### Live Attenuated Rubella, Measles, Mumps, and Varicella Vaccines

Rubella virus infection during pregnancy can lead to congenital rubella syndrome (CRS) as the virus has the ability to cross the placenta and infect the fetus. The risk to develop CRS is higher if rubella infection occurs in the first 12 weeks of gestation. Live attenuated rubella vaccines, licensed since the late 1960s and available for childhood immunization program in several countries, alone or in combination with measles, mumps and varicella vaccines, have lead to a notable reduction in the CRS cases (134).

As with the other LAVs, rubella vaccines are contraindicated in pregnant women, either alone or in combination with measles, mumps, and varicella.

Although wild-type rubella infection can be burdened by a higher risk of teratogenicity and intrauterine infection, inadvertent rubella vaccination during pregnancy or before conception has not been associated with a higher risk of adverse pregnancy outcomes such as congenital malformations, spontaneous abortion, prematurity, neonatal death, and low birth weight (136–138).

Of note, no CRS cases have been reported in a large study conducted in six Latin American countries between 2001 and 2008 on more than 2,800 pregnant women accidentally immunized with rubella vaccine, although anti-rubella specific IgM were detected in the cord blood of 3.5% of the newborns (139). The same is true for other studies in which no cases of CRS have been detected following accidental rubella immunization (140, 141). In a systematic review recently published by Mangtani et al. the maximum theoretic risk of CRS following accidental vaccination in pregnant women has been estimated to be 0.099% (134). Despite these data, vertical transmission of this attenuated vaccine virus has been detected (142) and some infants can develop a completely asymptomatic vaccine-virus infection (134).

Specific studies regarding the safety of mumps and measles vaccines in pregnant women are lacking, yet no evidence of a higher risk of congenital malformations and spontaneous abortion has been demonstrated (134). Similarly, no case of congenital varicella syndrome has been reported in a study conducted on 981 women inadvertently exposed to varicella vaccine (143).

At any rate, data from accidental vaccination during pregnancy is not sufficient to justify their use in pregnant women so that current guidelines contraindicate any live vaccine during pregnancy.

Table 1 summarizes maternal vaccines currently recommended, contraindicated and allowed in special circumstances during pregnancy.

**Table 1:** Vaccines recommended, contraindicated, and available in special circumstances during pregnancy.

**Vaccine**	**Type**	**Comments**	**References**
**Recommended**			
IIV (Inactivated Influenza Vaccine)	Inactivated	For all pregnant women at any time of gestation, as well as for all women planning to get pregnant or in the postpartum phase/breastfeeding during flu season.	(114)
Tdap (Tetanus, Diphtheria, Pertussis) vaccine	Inactivated	For all pregnant women during each pregnancy, regardless of personal history of previous vaccine, at 27–36 weeks of gestation (although it could be given at any time during pregnancy). Women who were not vaccinated during pregnancy should receive Tdap vaccine at postpartum.	(132)
**Available in special circumstances**			
Hepatitis A	Inactivated	In case of high-risk of infection (i.e., due to permanence in highly or intermediately endemic regions) the risk-benefit ratio should be considered.	(144)
Hepatitis B	Subunit	If case of high risk of infection (among risk factors: sexual promiscuity in the past 6 months, HbsAg-positive partner, intravenous drug abuse) to protect both mother and fetus.	(145)
Meningococcal ACWY	Inactivated	In case of high-risk of infection (i.e., due to close contact to affected patients, in addition to chemoprophylaxis, and to permanence in endemic/hyperendemic regions in specific immunocompromised hosts) the risk-benefit ratio should be considered.	(145)
Meningococcal B	Inactivated	In case of high-risk of infection (i.e., due to close contact to affected patients) the risk-benefit ratio should be considered. Main role of chemoprophylaxis.	(146)
Pneumococcal (PPSV23)	Inactivated	In case of high-risk of infection for women with certain chronic health conditions	(145)
Pneumococcal (PCV13)	Inactivated	In case of high-risk of infection, only when benefits outweigh risks (after consultation with patient's health care provider)	(145)
Japanese encephalitis	Inactivated	In case of high-risk of infection (i.e., before traveling to endemic regions, during outbreak) the risk-benefit ratio should be considered. Data on the safety, immunogenicity, and efficacy are scarce.	(147)
Tick- borne encephalitis	Inactivated	In case of high-risk of infection during outbreaks in endemic regions	(148)
Rabies	Inactivated	Post-exposure prophylaxis and pre-exposure in case of high-risk	(149)
Anthrax	Adsorbed	Post-exposure prophylaxis (plus 60 days of antimicrobial treatment)	(150)
Polio	Inactivated	In case of high-risk of infection (during outbreaks or before traveling in endemic regions) when the benefits outweigh the risks.	(151)
Cholera	Inactivated	In case of high-risk of infection (during cholera outbreaks and traveling in endemic regions)	(152)
Yellow fever	Live attenuated	Generally not recommended; in case of high-risk of infection (during outbreaks and for traveling in endemic regions) the risk-benefit ratio should be considered.	(153)
Typhoid	Inactivated	In case of high-risk of infection (during outbreaks, high exposure risk)	(146)
Small pox	Live attenuated	Fetus could be exposed to small but serious potential risk. The vaccine should not be administered in pregnant or periconceptual women except in case of high risk of contracting the disease, when the benefits outweigh the risks.	(146)
**Contraindicated**			
Measles-mumps-rubella	Live attenuated	Contraindicated during pregnancy. It could be administered during post-partum in rubella seronegative women. No case of Congenital Rubella Syndrome (CRS) has been detected after accidental vaccination in pregnant women.	(146)
Varicella	Live attenuated	Contraindicated during pregnancy. It could be administered during post-partum in varicella seronegative women. No case of Congenital Varicella Syndrome (CVS) has been detected after accidental vaccination in pregnant women.	(146)
Bacillus Calmette- Guèrin (BCG) vaccine	Live attenuated	Contraindicated during pregnancy.	(146)
Live zoster vaccine	Live attenuated	Contraindicated during pregnancy.	(146)
Live Attenuated Influenza Vaccine (LAIV)	Live attenuated	Inactivated vaccine available and recommended in pregnant women	(146)
Oral poliovirus vaccine	Live attenuated	Inactivated vaccine available	(146)
Typhoid live oral vaccine	Live attenuated	Inactivated vaccine available	(146)
HPV vaccine	Inactivated	Non-recommended. In case of incomplete immunization before pregnancy, it could be completed after delivery.	(146)

### COVID-19 Vaccination in Pregnancy

SARS-CoV-2 pandemic is the major global health emergency of our century.

The infection might lead to COVID-19 disease, described mainly by upper and lower respiratory symptoms, fever and in some cases by a systemic inflammatory syndrome, with high mortality (154). Pregnant women are also susceptible to the infection with a higher risk of preterm labor and pregnancy related complications (155). Little is known on the long-term consequences of the perinatal infection in the newborn.

As COVID-19 vaccination programs have been adopted by the states to deal with the health emergency, many concerns are rising regarding the safety of the licensed COVID-19 vaccines for pregnant women.

Two mRNA based-technology vaccines Pfizer/BioNTech and Moderna, and two viral vector vaccine-type, AstraZeneca and Johnson & Johnson, have been approved by most countries worldwide including European Union, United States and United Kingdom. Others are under rolling review.

Current recommendations suggest that pregnant women should be vaccinated when the benefits outweigh the potential risks (156). Thus, pregnant workers on the frontline and those with pre-existing conditions should receive the vaccine. About 20,000 pregnant women have been vaccinated for COVID-19 in USA and a smaller number in the UK (157, 158). The data is encouraging, as no major adverse effects have raised. Moreover, one case study described specific maternal IgG anti-spike protein in a newborn whose mother was previously vaccinated (159).

While awaiting more data about the risks and benefits of the vaccine during pregnancy, these encouraging studies suggest that maternal vaccination could provide newborns with some level of protection against infection.

## Future Directions

### Maternal Vaccines in Development

Beyond maternal vaccines currently available, several new vaccines are in development to protect against neonatal pathogens, including respiratory syncytial virus (RSV) and group B streptococcus (GBS).

#### Respiratory Syncytial Virus (RSV) Vaccination

RSV is the main cause of viral lower respiratory tract infections in children, especially in the first 2 years of age, leading to significant morbidity and mortality worldwide (160). It causes bronchiolitis and pneumonia and frequently results in hospitalization and recurrent wheezing or asthma (161).

While it is widely accepted that an effective vaccine against RSV would have a major impact on child health globally, few data are available on the incidence and impact of maternal RSV infection during pregnancy. A few observational studies have shown that RSV can cause acute upper and lower respiratory tract disease in pregnant women, particularly in the third trimester of gestation (162). However, being a respiratory virus, it may act like other viral respiratory infections in pregnancy, causing consistent morbidity of the mother (163). Therefore, using an effective RSV vaccine in pregnancy could potentially benefit both the mother and the baby.

The success and efficacy of passive immunization with palivizumab in infants, which bind to antigenic site II on the RSV F protein, has led many vaccine developers to focus on this as the primary immunogen (8). Several candidate vaccines are currently under clinical development (164). A phase 3, randomized, placebo-controlled trial investigated the safety and efficacy of RSV-F nanoparticle alum-adjuvanted vaccine. This trial is the largest study so far to evaluate a vaccine primarily designed for use in pregnant women (165). It has been conducted in 11 countries and involved 4,636 healthy pregnant women at 28–36 weeks 0 days of gestation (ClinicalTrials.gov identifier: NCT02624947). The results from this trial showed that maternal RSV F vaccine had an overall adverse event profile similar to placebo. Unfortunately, the primary endpoint of RSV-associated medically significant lower respiratory tract infection up to the first 90 days of life in infants was not met. However, a possible efficacy against RSV LRTI with severe hypoxemia and RSV LRTI with hospitalization was detected (48%vaccine efficacy, 95% CI: 8.2–75 and 44% vaccine efficacy, 95%CI: 19.6–61.5) (166).

At the moment, considering the high risk of RSV infection in neonates born prematurely, a combined approach with maternal immunization followed by administration of passive antibodies for protection against RSV in early life remains the optimal strategy for preterm infants at risk (167).

#### Group B Streptococcus (GBS) Vaccination GBS

GBS is an important cause of neonatal pneumonia, meningitis, and sepsis. The most common current strategy to reduce early-onset neonatal sepsis is screening for GBS in pregnant women and administration of intrapartum antibiotics to those who are colonized (168). However, the incidence of late-onset disease is not substantially reduced (169).

In addition, the identification and treatment of women at risk is often difficult in settings where access to diagnostic testing and intravenous antibiotics during labor is limited, as in many low and middle-income countries (170).

Thus, immunization of pregnant women with a GBS vaccine represents a potential strategy to protect newborns from invasive GBS disease through passive immunity.

Recently, the WHO developed a document titled “Group B Streptococcus Vaccine Development Technology Roadmap,” proposing priority activities for research, development and testing of GBS vaccines, to facilitate and accelerate vaccine licensure and guarantee global availability (171). One important goal of this effort is to conduct a pivotal efficacy trial with a vaccine that includes the most relevant GBS serotypes and specific, measurable, priority endpoints, such as the prevention of stillbirth, neonatal deaths, and late-onset GBS disease and its complications (167).

Several studies have been conducted in pregnant women, with monovalent (serotype III) and trivalent (serotypes Ia, Ib, III) conjugate vaccines against GBS. These vaccines demonstrated safety and tolerability and efficient transplacental antibody transfer to the infant. However, for the moment, none have yet entered Phase 3 development (8, 167).

### The Potential of Live-Attenuated Vaccines in Pregnancy

Live-attenuated vaccines (BCG, measles, mumps, rubella, smallpox, chickenpox, yellow fever) generally have the advantage of single-dose, rapid induction and durable immunity compared to inactivated vaccines. Epidemiological studies have also suggested that live vaccines extend protective effects toward unrelated pathogens (172). This unexpected pathogen-agnostic protection has been demonstrated for the measles, oral polio vaccine (OPV), BCG, and smallpox vaccines (3, 173–176). Heterologous immunity and trained immunity might favor the occurrence of an agnostic immunity that broadens beyond the specific immune response to vaccine antigens (177).

However, LAV are currently contraindicated during pregnancy because of the potential risk of causing infection and subsequent transplacental transmission of the pathogen to the developing fetus, recent systematic review and meta-analysis on maternal immunization safety with LAV mostly demonstrated no or very low risk of adverse pregnancy outcomes (135). Randomized clinical trials (RCTs) and non-RCTs studies should be performed to investigate the potential of vaccination with live vaccines in pregnancy as a tool to protect the mother and the newborn from infectious diseases.

### Challenges to Be Met With Maternal Vaccines

Although national and international health authorities have defined tetanus, pertussis, and flu maternal immunization schedules, vaccine uptake remains unsatisfactory. In fact, apart from few studies in the UK and US reporting a Tdap and flu uptake between 40 and 70% (refer to these studies), in most European countries vaccine coverage rarely reaches >10% (165). Several studies have investigated potential factors affecting pregnant women's awareness and behavior on seasonal flu and pertussis vaccine compliances (178). Different cultural, social, economic, and structural barriers need to be overcome (45, 179). Within our Italian project called VaxInPerMam, at Tor Vergata University Hospital, ~1,000 childbearing Italian women have been surveyed to evaluate flu and pertussis vaccine attitude and uptake. Less than 40% of women knew maternal immunization recommendations, and, mainly due to safety concerns, <10% would receive pertussis and flu vaccines (manuscript in preparation). Misinformation represents a key factor affecting vaccine uptake. Specific intervention programs directed at both the healthcare provider and the mother-to-be are required to overcome barriers and prejudices (180).

## Conclusions

Maternal immunological experience protects the child in the early postnatal period when the risk of infection is highest. Vertically transferred maternal antibodies of IgG isotype exert protection in the early postnatal period when the neonate has not yet been able to produce its own antibodies. Maternal IgA and IgG antibodies are also transferred through breastfeeding and increased levels of both isotypes can be detected in breastmilk after vaccination during pregnancy. Moreover, neonatal immune cells may be stimulated by maternal cells and antigens transferred *in utero*, with evidence of both activating and tolerogenic impacts on fetal immune components.

Maternal vaccination has already been shown protect the newborn from severe infections and currently represents the best defense option against various pathogens. The vaccines currently administered to pregnant women have excellent safety profiles. Strategies to overcome vaccine hesitancy include education on the importance of immunization in pregnancy through specific public awareness campaigns. Healthcare providers' recommendations are essential to increase vaccination coverage during pregnancy and for further advancement of a maternal immunization programs. Finally, a better understanding of the mother-child immunological link and of early life immunological steps will sustain further vaccine research and development to combat the surge of infectious diseases with benefit of the entire population.

## Author Contributions

BC, MC, MS, VM, and MD contributed to the conception and design of the work. BC, MC, and MS wrote the manuscript. GT, RE, RC, AF, EP, GB, MDC, AZ, VM, and MD revised it carefully for important intellectual content. All authors contributed to the manuscript revision, read, and approved the submitted version.

## Conflict of Interest

The authors declare that the research was conducted in the absence of any commercial or financial relationships that could be construed as a potential conflict of interest.
